# Comparison of Short-Term Results of Laparoscopic and Open Surgeries for Colorectal Cancer: A Single-Center Experience

**DOI:** 10.7759/cureus.24635

**Published:** 2022-05-01

**Authors:** Dogukan Durak, Ertugrul G Alkurt, Veysel Barış Turhan, Berksun Tutan, Ibrahim Tayfun Sahiner, Murat Kendirci

**Affiliations:** 1 Gastrointestinal Surgery, Hitit University Çorum Erol Olçok Training and Research Hospital, Çorum, TUR; 2 General Surgery/Surgical Oncology, Hitit Üniversitesi Erol Olçok Eğitim ve Araştırma Hastanesi, Çorum, TUR; 3 General Surgery, Turkish Ministry of Health Hitit University Çorum Erol Olçok Training and Research Hospital, Çorum, TUR; 4 General Surgery, Hitit University Çorum Erol Olçok Training and Research Hospital, Çorum, TUR; 5 General Surgery, Hitit University, Faculty of Medicine, Çorum, TUR

**Keywords:** lymph node metastasis, open surgery, laparoscopic surgery, oncological surgery, colorectal cancer

## Abstract

Objective: Although laparoscopic colon cancer surgeries have increased in recent years, their oncological competence is questioned. In our study, we aimed to evaluate oncological competence by comparing laparoscopic and open surgery.

Method: The study was planned retrospectively. A total of 94 patients were included in the study, 42 of whom underwent laparoscopy, and 52 patients underwent open surgery. Both groups were compared in terms of demographic characteristics, staging, number of benign/malignant lymph nodes, histological findings, and complications.

Result: The final pathology report of all patients was adenocarcinoma. The median number of dissected lymph nodes was 20.9 in the open group (8-34) and 19.46 in the laparoscopy group (7-31) (p = 0.639). The median number of dissected malignant lymph nodes was 1 (0-13) in the open surgery group and 3.1 (0-8) in the laparoscopy group (p = 0.216). The laparoscopy group exhibited a longer operation time (281.2 ± 54.2 and 221.0 ± 51.5 min, respectively; p = 0.036) than the open surgery group, but a shorter intensive care unit (ICU) discharge, quicker initiation oral feeding, and shorter length of hospital stay (4.0 ± 0.9 vs 5.7 ± 2.0 days, respectively; p < 0.001).

Discussion: Laparoscopic surgery elicits many benefits such as less wound infection, lower requirement for blood transfusion, shorter hospitalization, quicker initiation of oral feeding, and mobilization. Our study has shown that laparoscopic surgery provides quite adequate lymph node dissection when compared to oncological surgery, which is viewed with suspicion in the light of these benefits of laparoscopy.

## Introduction

Colorectal cancer is the third most common cancer worldwide and ranks fourth in cancer-related deaths [1]. Surgery still stands as the gold standard in the treatment of colorectal malignancies to provide a complete, customized, and final treatment. The first series of laparoscopic colorectal surgery were reported in the United States in 1991 [2]. Later, laparoscopic interventions for colorectal cancer, appendicitis, and diverticulitis were included in the list of indications [3]. Although in 1994, some authors claimed that laparoscopic colorectal surgery is contraindicated due to trocar site tumor growth, it is not accepted today [4]. The study of Berends et al. [5] reported port-side recurrence as 21%; however, some available studies are reporting this rate below 1% [6].

In today's world, laparoscopic surgery is a widely used procedure. Therefore, clinical trials were conducted to compare short-term and long-term survival outcomes with those of open surgery, and laparoscopic surgery was proven to be a superior option to open surgery in terms of length of hospital stay, surgical complications, faster recovery, return to work, and aesthetic and immunological effects [7-9]. In the light of these studies, laparoscopy was found to be safe and efficient to use in cases of colorectal cancer [10-13]. However, experts are still investigating whether laparoscopic surgery follows oncologic principles and whether oncologic outcomes are equivalent to those of open surgery.

Also, laparoscopic colorectal surgery has not received widespread approval and is not considered the gold standard treatment globally. Patients who had laparoscopic surgery or open surgical resection for colorectal cancer had short-term histological outcomes and complication rates compared to this study.

## Materials and methods

The research comprised patients who were diagnosed with colorectal cancer and had surgery at the Hitit University Hospital between December 2019 and October 2021. A total of 42 patients who underwent laparoscopic surgery were included in the study. Additionally, 52 patients with similar age and gender distribution who underwent open surgery were randomly selected.

Patients with a body mass index above 30 kg/m2, distant metastases, synchronous tumors, and patients who were operated on for mechanical bowel obstruction were excluded from the study. All procedures were performed by an experienced colorectal surgical team. A total of 94 patients meeting the criteria were included in the study.

Ethics committee approval was received from the Hitit University Ethics Committee in 2022 (Ethics Committee Decision Number: 2022-14). The study was conducted in compliance with the Declaration of Helsinki. Patients whose data were completely accessible and who fit the research requirements were added after a retrospective examination of the clinical archive system.

Colorectal cancer diagnosis and synchronous tumor detection were confirmed by colonoscopy and colonoscopic biopsy. The participants in the research were separated into two groups: those who underwent laparoscopic surgery and those who underwent open surgery. Thoracic and abdominal tomography was routinely performed on all patients to determine whether distant metastases were present. Preoperative bowel cleansing was routinely performed in all patients. All patients received prophylactic antibiotics and anti-thromboembolism drugs. Open and laparoscopic colorectal surgery procedures were performed according to previously published standard protocols. Both groups were compared in terms of demographic characteristics, staging, number of benign/malignant lymph nodes, histological findings, and complications. The seventh edition of the American Joint Committee on Cancer was used for staging.

Statistical analysis

Statistical analysis was performed using IBM SPSS (Statistical Package for the Social Sciences) Statistics for Windows, Version 22.0 (IBM Corp., Armonk, NY). The age variable was reported as mean SD and analyzed with a t-test. Normally distributed data were analyzed by the t-test. The Mann-Whitney U test was used to analyze non-normally distributed data, expressed as median and range. The Shapiro-Wilk test was used to determine data normality. A p-value of below 0.05 was considered statistically significant.

## Results

Open surgery (n = 52, 55.4%) and laparoscopic surgery (n = 42, 44.6%) were performed in a total of 94 patients. The final pathology report of all patients was adenocarcinoma. While a total of five patients had poorly differentiated adenocarcinoma, 42 patients had moderately differentiated adenocarcinoma and 47 patients had well-differentiated adenocarcinoma. In the open surgery and laparoscopic groups, the number of stage 1 patients was 11 (10.3%) and five (11.9%), respectively, while 21 (38.8%) and 19 (45.2%) patients were diagnosed as stage 2, and 22 (40.7%) and 18 (42.8%) patients were diagnosed as stage 3 (p = 0.682).

The median number of dissected lymph nodes was 20.9 in the open group (8-34) and 19.46 in the laparoscopy group (7-31) (p = 0.639). The median number of dissected malignant lymph nodes was 1 (0-13) in the open surgery group and 3.1 (0-8) in the laparoscopy group (p = 0.216) (Table 1). Blood transfusion was required in 10 patients in the open surgery group and seven patients in the laparoscopy group (p = 0.256). Anastomotic leakage was observed in a total of three patients, two of which were in the open surgery group and one was in the laparoscopy group (p = 0.456). The laparoscopy group exhibited a longer operation time (281.2 ± 54.2 and 221.0 ± 51.5 min, respectively; p = 0.036) than the open surgery group but a shorter ICU discharge, quicker initiation of oral feeding, and shorter length of hospital stay (4.0 ± 0.9 vs. 5.7 ± 2.0 days, respectively; p < 0.001) All operations were successfully completed without damaging the adjacent organs or blood vessels.

**Table 1:** Patient demographic features and pathological characteristics of the tumors *Statistically significant data. TNM, tumor-node-metastasis; T, tumor; N, node; SSI, surgical site infection; SD, standard deviation; LAP, lymphadenopathy; LP, laparoscopy.

	Open	LP	p-value
Number of patients, n (%)	52 (55.4)	42 (44.6)	
Age, years, Mean ± SD	64.25 ± 9.56	65.87 ± 12.75	0.559
Gender n (%)			0.211
Female	22 (42.4)	20 (47.6)	
Male	30 (57.6)	22 (52.4)	
Pathology n (%)			0.831
Low differentiated adenocarcinoma	3 (5.7)	2 (4.7)	
Moderately differentiated adenocarcinoma	20 (38.4)	20 (47.6)	
High differentiated adenocarcinoma	22 (42.3)	20 (47.6)	
Final stage n (%)			0.682
1	11 (10.3)	5 (11.9)	
2	21 (38.8)	19 (45.2)	
3	22 (40.7)	18 (42.8)	
T stage n (%)			0.565
T1	2 (3.8)	2 (4.7)	
T2	6 (11.5)	8 (19)	
T3	42 (80.7)	31 (73.8)	
T4	2 (3.8)	1 (2.3)	
N stage n (%)			0.136
N0	25 (48)	18 (42.8)	
N1	26 (50)	(52.3)	
N2	1 (2)	2 (4.7)	
Number of LAP excessed, median (min-max)	20.9 (8-34)	19.46 (7-31)	0.639
Malignant LAP excessed, median (min-max)	1 (0-13)	3.1 (0-8)	0.216
Complications, n (%)			
İleus	6 (11.5)	5 (14.2)	0.485
SSI	6 (11.5)	1 (2.3)	0.032*
Leak	2	1	
Blood transfusion	10 (19.2)	7 (16.6)	0.256
Hospital stay, mean, day	10.4 ± 6.8	7.1 ± 4.9	0.048*

A total of 21 patients developed early postoperative complications. A comparison of early complication rates revealed that postoperative ileus occurred in six patients in the open group, while ileus occurred in five patients in the laparoscopy group. There was no statistically significant difference (p = 0.485). However, wound infection was observed in six patients undergoing open surgery and only one patient undergoing laparoscopy (p = 0.032). The length of hospital stay was also statistically shorter in the laparoscopy group.

## Discussion

Laparoscopic surgery is currently being used in oncological situations with excellent results. This study has shown that laparoscopic surgery provides quite adequate lymph node dissection when compared to oncological surgery, which is viewed with suspicion in the light of these benefits of laparoscopy. We think that laparoscopy, following a sufficient learning curve, is as advantageous and safe in malignant diseases, as in benign diseases.

Colorectal cancer incidence is on the increase as a result of genetic predisposition, advanced age, environmental factors, and lifestyle [14]. With the developing technology, minimally invasive approaches come to the fore in the treatment of colorectal cancer. Despite the many accepted benefits of the laparoscopic approach for colorectal cancer, its oncological adequacy is still questioned. In our study, we compared single-center laparoscopy and open surgery for colorectal cancer, taking into account all the advantages and disadvantages, especially with oncological competence in the foreground.

In the study of Kitona et al., laparoscopic and open colorectal surgery were compared; in this study, they observed that laparoscopy resulted in a shorter hospital stay, faster recovery, and less site infection [15]. In the COREAN (Comparison of Open versus laparoscopic surgery for mid or low REctal cancer After Neoadjuvant chemoradiotherapy) and COLOR II (COlorectal cancer Laparoscopic or Open Resection) studies, it was observed that the length of hospital stay was one day shorter in laparoscopy [16,17]. In addition, two similar studies reported that hospital stay was seven days in laparoscopic surgery, while it was eight days in open surgery [18-20]. In our study, the mean length of hospital stay was 10.1 days in open surgery, while it was seven days in laparoscopic surgery, which was consistent with the literature.

In a study that included a meta-analysis of 3,410 patients, it was observed that the operation time was shorter in open surgery, whereas the length of hospital stay was shorter in laparoscopy [21]. Although the duration of open surgery was shorter than laparoscopic surgery in our study, there was no statistical difference. According to the same meta-analysis, no statistical difference was found between the two groups in terms of anastomotic leakage and postoperative ileus [21].

In our study, leakage occurred in two patients in open surgery and one patient in laparoscopic surgery, but there was no statistical difference. Furthermore, we observed postoperative ileus in six patients undergoing open surgery and five patients undergoing laparoscopic surgery, but there was no statistical difference. However, as shown by the same meta-analysis and supported by our study, wound infection was more common in open surgery. This is due to larger incisions in open surgery and a greater risk of contamination during resection and anastomosis.

A meta-analysis of 4,747 patients reported a lower requirement for blood transfusion in laparoscopy than in open surgery [22]. In the study conducted by Sheng et al., no difference was observed between the two groups in terms of the requirement for blood transfusion [23]. Consistently, the requirement for blood transfusion was lower in the laparoscopy group in our study, but there was no statistical significance. This may be due to the small number of grouped patients.

Referring to the oncological results in which actual laparoscopic surgery is investigated, the study of Tong et al. found no difference between laparoscopy and open surgery in lymph node dissection [22]. Although the number of lymph nodes was higher in the laparoscopy group in the study of Yang et al., no statistically significant difference was observed [24]. In the study of Liang et al., no significant difference was found between the open and laparoscopic surgeries in terms of proximal, distal, and radian surgical margins and the number of lymph nodes dissected [25].

Shuai-Xi Yang compared robotic, laparoscopy, and open surgeries and reported that robotic surgery was superior in lymph node dissection, but no statistically significant difference was observed between the laparoscopy and open surgery [26]. In our study, a mean of 20.9 lymph nodes was dissected in open surgery, whereas a mean of 19.46 lymph nodes was dissected in laparoscopy, and no statistically significant difference was observed. Although our results are consistent with the literature, no oncological difference was observed between the open and laparoscopic surgeries. This may be attributed to efficient visualization and dissection of both vessels and lymph nodes, thanks to the high-resolution image quality in laparoscopic surgery. Moreover, a higher number of dissected lymph nodes in robotic surgery supports our thesis.

Although the results of our study are consistent with the literature, the fact that it is single-centered and has a relatively low number of patients may be a limiting factor. Additionally, we think that increased quality of the technical equipment in practice can especially improve the results of oncological surgery.

## Conclusions

As a result, laparoscopic surgery is a surgical procedure with many advantages to oncological colon surgery. It has the advantages of less hospital stay and fewer complications. However, it was seen in the study that it is not different from open surgery in terms of oncology, and adequate surgical margin and lymph node dissection can be achieved. We think that laparoscopic surgery in colon cancers is safe in terms of oncology.
